# Feasibility and acceptability of an enhanced cognitive behavioural therapy programme for parent–child dyads with anxiety disorders: a mixed-methods pilot trial protocol

**DOI:** 10.1186/s40814-021-00846-8

**Published:** 2021-05-17

**Authors:** Samantha Galea, Chloe A. Salvaris, Marie B. H. Yap, Peter J. Norton, Katherine A. Lawrence

**Affiliations:** 1 Turner Institute for Brain and Mental Health, School of Psychological Sciences, Monash University, Level 4, Bldg 18 Innovation Walk, Clayton, Victoria 3800 Australia; 2 Melbourne School of Population and Global Health, University of Melbourne, Carlton, Melbourne, Victoria Australia; 3 Cairnmillar Institute, Hawthorn East, Melbourne, Victoria Australia

**Keywords:** Cognitive behavioural therapy, Anxiety disorders, Parent and child, Feasibility, Acceptability, Pilot trial, Mixed-methods

## Abstract

**Background:**

Cognitive behavioural therapy (CBT) is the most widely recognised and efficacious psychological therapy for the treatment of anxiety disorders in children and adults. However, suboptimal remission rates indicate room for improvement in treatments, particularly when both children and their parents have anxiety disorders. Bidirectional transmission and maintenance of anxiety within parent–child dyads could be better targeted by CBT, to improve treatment outcomes for children and parents with anxiety disorders. This study aimed to develop and evaluate the feasibility and acceptability of a concurrent parent–child enhanced CBT intervention that targets the individual’s anxiety disorder(s), as well as the bidirectional factors that influence and maintain anxiety in the dyad.

**Methods:**

Feasibility and acceptability of the proposed CBT protocol will be evaluated in an open-label pilot trial of the intervention utilising qualitative and quantitative data collection. Ten parent–child dyad participants (*n* = 20) with anxiety disorders will be recruited for the proposed intervention. The intervention is based on an empirically supported 10-week CBT programme for anxiety disorders in adults, adapted to be delivered to parent–child dyads concurrently, and to target anxious modelling and overprotective behaviours through joint observational exposures. Intervention feasibility will be explored by pre-post symptom change on a range of clinician- and self-report measures to determine preliminary indications of participants’ intervention response and effect size calculations to estimate sample size for a future definitive randomised controlled trial (RCT). Additional feasibility measures will include recruitment rates, completion rates, and adherence to programme requirements. To explore participant acceptability of the intervention, qualitative interviews will be conducted with five parent–child dyads who complete the intervention (*n* = 10), along with five parent–child dyads with anxiety symptoms who express interest in the intervention (*n* = 10). Acceptability measures will include prospective and retrospective quantitative self-report and qualitative interview data.

**Discussion:**

This pilot trial will utilise a mixed-methods design to determine the feasibility and acceptability of delivering an enhanced CBT intervention for the concurrent treatment of parent–child dyads with anxiety disorders. The results of this trial will inform the development and implementation of a future definitive randomised clinical trial to evaluate intervention efficacy.

**Trial registration:**

Australian and New Zealand Clinical Trials Registry, ANZCTR1261900033410. Prospectively registered: pre-results. Registered 04 March 2019.

## Background

Anxiety disorders are amongst the most prevalent class of mental health disorders in adults and children [1, 2]. They often first occur in childhood and continue into adulthood if untreated [3]. Across the lifespan, the presence of clinical anxiety is associated with poorer outcomes in interpersonal, academic, occupational, and health domains [4, 5]. Cognitive behavioural therapy (CBT) is recognised as the most efficacious and cost-effective psychological treatment for anxiety disorders in adults and children [6–8]. Despite its extensive evidence base, recent meta-analytic results of CBT for anxiety disorders reported mean remission rates of approximately 50% in adults and children [9, 10]. These modest response rates suggest significant scope for improvement of treatment outcomes [6, 11]. Given the prevalence of anxiety disorders and associated negative impacts for individuals and society [4, 12, 13], further research refining and trialling efficacious CBT protocols targeting anxiety is warranted.

The link between parental anxiety and the development and maintenance of anxiety disorders in children is widely reported. Several clinical trials of CBT for treating child anxiety disorders have found that having a parent with clinical levels of anxiety can significantly reduce CBT effectiveness, as assessed by pre-post clinician- and self-report measures of anxiety severity [14–18]. In addition to parental psychopathology, various parenting factors have been proposed to mediate parent–child anxiety transmission, and contribute to poorer treatment outcomes in children. These parenting factors include rejection, overprotection, accommodation, parent cognitions of child competence, modelling of anxiety, and information transfer [19–21]. Of these factors, parental modelling of anxiogenic responses and overprotective parenting behaviours have been most consistently associated with child anxiety [22, 23], with growing evidence emerging for the role of parental accommodation in maintaining the child’s anxiety [24]. Specifically, parents with anxiety are more likely to model avoidance and show increased sensitivity to child distress and increased apprehension when their child engages in age-appropriate tasks [25]. When repeatedly exposed to parents’ anxious responses, children may vicariously learn to respond in similarly anxious ways [26, 27]. Overprotective parenting increases children’s risk of developing anxiety disorders [22], by reducing opportunities to develop self-confidence and adaptive coping behaviours in new or challenging situations [28–30]. This in turn maintains the anxiety, as children form negative cognitions of being unable to cope, and increasingly avoid threatening stimuli [30, 31].

While less is known about the impacts of child anxiety on parent anxiety treatment outcomes, a growing body of research suggests that the presence of child anxiety can impact on anxiety in parents. A study investigating interactions in mother–child dyads indicated that anxious and non-anxious mothers of anxious children expressed greater catastrophising cognitions compared to anxious and non-anxious mothers of children without anxiety disorders [32]. Other studies investigating bidirectional anxiety relationships in parent–child dyads found that post-treatment reductions of children’s anxiety symptoms were associated with later reductions in parental anxiety [33], and overprotective and controlling parenting behaviours [34]. Similarly, other research has indicated that higher child anxiety predicted greater parental control [35], over involvement [36, 37], and parental accommodation [38]. Taken together, this body of research indicates that the presence of anxiety in a child, irrespective of their parent’s psychopathology, may influence behavioural responses and anxiety symptoms in parents.

Despite evidence for bidirectional influences of anxiety disorders in parent–child dyads, most prior research has typically involved parents as co-facilitators in child anxiety treatment [39–41] but has neglected to adequately address anxiety in parents [42]. However, there have been three notable exceptions to date which have examined whether targeting anxiety in parents might improve child CBT outcomes. In two randomised controlled trials (RCT) of CBT for child anxiety disorders, Cobham et al. [17] and Hudson et al. [14] included an adjunct parental anxiety management (PAM) programme to treat co-occurring parental anxiety. While results of the Cobham study indicated PAM initially improved diagnostic outcomes for children with an anxious parent compared to control, significant differences in treatment outcome were not maintained at 6- and 12-month follow-up. Similarly, Hudson reported that the addition of PAM conferred non-significant differences in remission rates for child primary anxiety diagnosis compared to child-CBT alone at post-treatment and 6-month follow-up. Furthermore, adjunct PAM did not improve self-reported anxiety symptoms [17] or remission rates for parents with an anxiety disorder [14] at post-treatment or follow-up. These findings suggest that the brief PAM sessions were insufficient for treating parent psychopathology. An evidence-based treatment such as CBT may be required to target parental anxiety, to in turn improve CBT outcomes for children with anxiety disorders.

The third study was a comprehensive three-arm RCT where children received CBT and their mothers were assigned to either maternal-CBT conducted 8 weeks prior to their child’s CBT, a concurrent mother–child interaction (MCI) intervention, or maternal active control [20]. The MCI intervention was designed to reduce potentially anxiogenic dynamics within the parent–child relationship and to increase maternal autonomy-promoting cognitions and behaviours. The maternal active control condition involved sessions which promoted family physical health behaviours. Results indicated the addition of maternal-CBT, or MCI intervention did not significantly improve outcomes for children beyond child-CBT alone. Additionally, mothers treated with CBT initially showed greater primary anxiety disorder remission than mothers in the MCI or active control conditions; however, this effect was no longer significant after children received CBT. Maternal-CBT results were not maintained following child-CBT as reductions in child anxiety led to reductions in mothers’ anxiety symptoms across conditions. While this result supports the bidirectional influence of child anxiety, the reported primary anxiety disorder remission rates for mothers across conditions ranged from 39.4 to 57.8%, consistent with meta-analytic adult CBT remission rates [9]. Therefore, a further scope for the improvement of parent treatment outcomes exists. Additionally, while the inclusion of evidence-based treatment for anxiety in parents takes a positive direction, the non-significant differences between treatment arms suggest that treating mothers 8-weeks prior to children may not have adequately intervened with bidirectional factors maintaining anxiety disorders within the dyad. Furthermore, since anxiogenic parent–child interactions are known risk factors for child anxiety [22, 26, 27] and appear to contribute to the bidirectional nature of anxiety maintenance within the dyad [32–34], targeting anxiogenic interactions *concurrently* with CBT for child and parent anxiety may achieve stronger effects for parent and child anxiety treatment outcomes.

While substantial evidence indicates bidirectional associations maintain anxiety in parent–child dyads, the impact of these bidirectional factors has not been adequately catered for by existing treatment protocols. Hence, we propose a treatment approach that targets anxiety symptomatology in both parents and children, as well as bidirectional factors which maintain and exacerbate anxiety disorders within the parent–child dyad via graded exposure techniques. The proposed protocol is adapted from an existing transdiagnostic CBT manual [43], which has demonstrated successful pre-post treatment outcomes in several previous clinical trials [44–46]. This manual was chosen to facilitate a concurrent treatment format for children and parents regardless of their specific anxiety diagnoses. To ensure alignment of treatment approaches and consistent session content for both members of the dyad, the original adult protocol was modified for child participants rather than utilising an existing child CBT protocol for anxiety. The process of adapting the original protocol for children involved extensive developmentally appropriate translations based on knowledge of the cognitive and emotional developmental stages of the target treatment population. Additionally, feedback on a preliminary version of the adapted protocol was provided by primary school-aged children to ensure that content was ‘child-friendly’ and comprehensible. Subsequently, the following major adaptations and additions to the original protocol were made: (1) the development of a child-version of the adult protocol, (2) addition of psychoeducation and cognitive strategies for parents targeting bidirectional factors which maintain anxiety within the dyad, and (3) incorporation of joint observational exposure sessions.

The joint observational exposures are considered the key component of the proposed intervention and to our knowledge has not been undertaken as a component of CBT previously. In the joint observational exposure activities, the parent will undertake an individual exposure while being observed by the child. Following this, the child will participate in an exposure activity while the parent observes. Neither dyad member will be directly involved in the other’s exposure; however, after acting as observers, both the parent and child will complete post-exposure reviews separately with their individual therapist to reflect on new evidence that emerged from observing the exposures.

Grounded in Bandura’s social learning theory [47] and the recognised impacts of parental modelling on anxiety transmission in their children [19, 36, 48], joint exposures allow parents to model alternative adaptive coping behaviours to their child. Through observation, it is expected that the child will vicariously learn that experiencing anxiety is normal and manageable, thus increasing perceptions of self-efficacy and coping ability. Additionally, as parental anxiogenic cognitions are both a known risk factor for anxiety in children and a maintenance factor for anxiety in parents [32, 36], by observing their child, parent cognitions about the child’s coping ability and resilience may be modified. It is subsequently anticipated that modified parent cognitions will lead to reductions in overprotective and accommodating behaviours. Furthermore, as silent observers, parents practice regulating their own physiological anxiety response and behavioural impulses to overprotect. We anticipate that this adapted exposure approach will significantly improve treatment outcomes for parents and children with anxiety disorders.

In line with guidance from the Medical Research Council on the development and implementation of complex intervention [49], the current study is an initial step in determining if the proposed enhanced intervention is feasible and acceptable for parent–child dyads with anxiety disorders. A mixed-methods approach was chosen to explore preliminary indications of participants’ intervention response and to include the essential contribution of the client voice, which provides a richer understanding of participants’ programme acceptability [50]. In accordance with the CONSORT extension for pilot and feasibility trials [51], trial feasibility asks questions related to whether a future trial can be done, should be done, and how. To address feasibility, the present study includes a calculation of effect size estimates for outcome measures to estimate the sample size of a future definitive RCT [51]. Further, feasibility will be assessed via recruitment rate, completion rate, and adherence to study requirements. Trial acceptability will be examined within the theoretical framework of acceptability as outlined by Sekhon et al. [52]. This framework defines acceptability as consisting of multiple components that reflect clients’ anticipated (prospective) or experiential (retrospective) responses to an intervention [52]. The seven component constructs outlined in the framework include affective attitude, perceived effectiveness, burden, self-efficacy, ethicality, intervention coherence, and opportunity costs [52]. Outcomes of the pilot feasibility and acceptability trial will inform whether to proceed with a powered randomised controlled trial to determine treatment efficacy and intervention validation in the future.

To the best of our knowledge, the proposed protocol is the first to concurrently treat parents and children with anxiety disorders, and to utilise a joint observational exposure format. The overarching aims of this study are as follows:

1. Assess study feasibility through investigation of:
  - Variability in clinician-report and self-report outcome measures to determine preliminary indications of participants’ intervention response;
  - Effect size calculations to estimate the sample size of a future definitive RCT;
  - Percentages of recruitment rate, completion rate, and adherence to study requirements.
2. Explore participants’ prospective and retrospective acceptability of the enhanced programme using quantitative self-report and qualitative interview data collection methods.

## Method

### Study design and setting

Feasibility and acceptability of the protocol will be evaluated utilising a mixed-methods design, encompassing qualitative and quantitative data collection. The intervention component is an open-label pilot trial (hereafter referred to as 'the intervention'), utilising a pre-test–post-test within-groups design to obtain an effect size estimate for future RCT planning. The intervention will be conducted in person at the Monash University psychology training clinic. Acceptability of the protocol will also be investigated with qualitative interviews of parent and child dyads. All qualitative interviews will be conducted remotely via video conferencing software.

### Participants and sample size

The intended intervention sample will be 10 parent–child dyads (*n* = 20). This sample size is recommended by Birkett and Day [53] as sufficient for a pilot feasibility trial effect size estimate in advance of conducting a powered RCT. Parent participants will be adults 18 years and older, while child participants will be aged 6–12 years. The parent participant is defined as the biological parent and/or primary caregiver of the child participant. For the qualitative interviews, approximately 10 parent–child dyads (*n* = 20) will participate; however, a slightly higher number of dyads may be recruited to achieve data saturation. Purposive sampling will be utilised to gather a diverse range of consumer perspectives. Therefore, participants will include five dyads (*n* = 10) that have completed the intervention to determine retrospective acceptability, and five dyads (*n* = 10) that express interest in participating to evaluate prospective acceptability.

### Eligibility criteria

#### For the intervention

Parent–child dyads (child age range 6–12 years) must both meet criteria for a primary diagnosis of an anxiety disorder as defined by the *Diagnostic and Statistical Manual*, 5^th^ edition (DSM-5) [54]. Clinician-administered diagnostic interviews will be undertaken prior to the intervention phase of the study to assess for current primary anxiety disorder diagnoses in both dyad members (see ‘Pre-intervention assessment’, below). Dyads who present with a principal DSM-5 diagnosis other than an anxiety disorder, any other condition of sufficient severity that requires immediate clinical prioritisation, e.g. suicidality and neurocognitive impairment, or insufficient English ability, will be excluded from the study and referred to appropriate alternative services.

#### For the qualitative data

As participants who complete the study protocol have previously met intervention eligibility criteria, the only additional requirement is the completion of the intervention. For parent–child dyads who express interest in the intervention, additional eligibility requirements are that they self-report current anxiety symptoms and have not previously participated in the intervention. Exclusion criteria for the qualitative study are the same as those specified for the intervention.

### Recruitment and screening

Potential participants will be recruited via promotional study flyers sent to local schools for dissemination amongst school mental health and wellbeing staff, and publication in school newsletters. Additionally, paid targeted advertising on social media will be utilised. Promotional material will contain a link to an electronic expression-of-interest form. Interested parents will be contacted by study researchers to complete a brief phone screening. For the intervention, potentially eligible parent–child dyads will be invited to participate in a formal diagnostic pre-intervention assessment. Ineligible families as indicated by the phone screening will be provided with information to access alternative psychological support, if required. For the qualitative interviews, dyads who previously participated in the intervention or who had expressed interest in being involved will be invited to participate. Following phone screening, eligible families will submit online informed consent prior to their interviews.

### Pre-intervention assessment

Prior to conducting the pre-intervention assessment, parents and children will be provided with detailed age-appropriate explanatory statements, and informed consent and assent to participate will be obtained. The Anxiety Disorders Interview Schedule for DSM (ADIS-5) [55] for parents and the Anxiety Disorders Interview Schedule for DSM Child and Parent Versions (ADIS-IV-CP) [56] for children will be administered to determine presence of a primary anxiety disorder diagnosis. The adult and child versions of the Columbia-Suicide Severity Rating Scale (C-SSRS) [57] will be administered to parents and children respectively, to determine risk status. Following the diagnostic interviews, parent–child dyads will be invited to attend a feedback session to discuss assessment results and treatment recommendations. Ineligible dyads will be presented with options for referral and/or alternative treatment recommendations. Eligible dyads will be invited to participate in the intervention.

### Qualitative interview procedure

All interviews will be conducted via video conferencing software at times convenient to families. The parent interviews will be conducted in two parts, with part 1 (parent focus) administered by SG and immediately followed by part 2 (child focus) administered by CS. Child interviews will be undertaken separately to the parent interview, by CS. It is anticipated that children will be interviewed independently of their parent; however, parents will be permitted to sit in on the interview if requested. Parents who elect to sit in on child interviews will be asked not to provide input to interview content. Parent interviews are estimated to take 60–90 min; child interviews will be approximately 30 min. For dyads who participated in the intervention, interviews will be conducted following the conclusion of the intervention. Dyads who have not participated in the intervention will be interviewed as they are recruited. All dyads will receive a $30 gift card as reimbursement for their time participating in the interviews.

### Intervention

The concurrent intervention will consist of ten treatment sessions. Sessions will be conducted individually, with one therapist, although some intervention activities will be shared experiences involving the dyad and two therapists. Sessions will occur weekly and last for 60 min each. Intervention content follows a typical CBT format of psychoeducation, cognitive restructuring, exposure, and relapse prevention, with homework assigned and reviewed weekly (see Table 1 for intervention content outline). The following major adaptations and additions to the original protocol [43] were made to tailor the intervention for parent–child dyads:

1. Child-version of the adult protocol: modifications included extensive changes in language and simplification of some CBT concepts to accommodate the developmental stage and cognitive abilities of the target participant age group. To increase programme appeal, vignettes with child-relevant content, diagrams, and illustrations; a workbook with colourful worksheets and handouts; and a rewards chart to monitor and acknowledge progress were included in the child treatment manual. Appropriate rewards for completing treatment components will be individually negotiated between the parent and child.
2. Additional psychoeducation for parents on bidirectional factors: specifically, psychoeducation on the impact of overprotection, accommodation, and modelling anxious behaviours on child anxiety. These modifications are illustrated by vignettes specific to the parent–child dyad.
3. Content modifications to address anxiogenic cognitions related to the parenting role: cognitive restructuring tasks to identify bidirectional anxiety maintenance factors and normalise anxiogenic cognitions in the dyad.
4. Joint observational exposure sessions: during joint observational exposures, dyad members act as silent observers of each other’s exposure. For example, a child may observe his parent with social anxiety disorder initiating a conversation with a stranger, while a parent may observe her daughter with selective mutism read a page of a storybook aloud to the therapist. For parents, cognitive restructuring is undertaken prior to observing their child. This includes identifying cognitions about their child’s expected performance and coping ability, and the parent’s ensuing overprotective response. Following joint observational exposures, both dyad members’ complete post-exposure reviews to identify when the other member successfully modelled adaptive coping behaviours, and to reflect on new evidence to challenge prior anxious assumptions.

**Table 1:** Intervention content

Session	Cognitive behavioural therapy strategies and description
Session 0: ‘Pre-Treatment Feedback Session’	Present and discuss anxiety assessment results with participant^a^ (parent is present for child assessment feedback). Familiarise the participants with the treatment structure. Describe and illustrate subjective units of distress ratings and coping response. Set treatment goals by developing a trigger response hierarchy.
Session 1: ‘Introduction to the Anxiety Treatment Program’	Psychoeducation to create shared understanding of terminology and to normalise the experience of anxiety. Psychoeducation on: The development and maintenance of anxiety disorders and the relationship between thoughts, feelings and behaviours. Child only: introduction to the workbook, achievement charts, and rewards. Parent only: psychoeducation on modelling of anxiety.
Session 2–Session 3: ‘Challenging and Changing our Anxious Thoughts’	Additional psychoeducation on the importance of thoughts as antecedents to anxiety. Introduction to the concept of cognitive restructuring. Participants will learn to identify anxious thoughts, recognise cognitive biases and assumptions through the utilisation of challenging questions, and generate adaptive alternative responses to reduce habitual anxious cognitions. Parent only: psychoeducation on bidirectional nature of anxiety in parent–child dyads, overprotective and accommodating parenting behaviours.
Session 4–Session 6: ‘Facing Our Fears Together’	Participants undertake exposure activities to systematically confront items on their trigger-response hierarchies^a^. Parent and child dyads observe each other completing exposures. Exposures function to habituate clients to the physiological responses of anxiety, provide a learning opportunity to evaluate the validity of catastrophic fears, and reduce avoidance/escape behaviours. Cognitive restructuring and review is completed pre- and post-observing and participating in exposure tasks.
Session 7–Session 9: ‘Facing Our Fears’	Participants continue individual exposure activities. Sessions follow the same format as previous exposure sessions but without the observational component. Parent only: parent’s anxiety triggers that may be distressing to children will be targeted during these sessions.
Session 10: ‘Finishing Treatment: Where to From Here?’	Relapse prevention including treatment review, psychoeducation, and skills development on maintaining successes and continuing to make progress and managing setbacks. Congratulations and celebration of treatment completion^a^.

*Note.* Parents and children are treated in separate individual treatment sessions, although some treatment activities are shared experiences

^a^Shared treatment activities

### Data collection

Participants will complete self-report feasibility and acceptability measures using paper versions of all questionnaires. Verbal assistance may be provided by clinicians to enhance child participants’ comprehension of self-report questionnaires, if required. Administration time-points for all clinician assessments and self-report measures are listed in Table 2. 
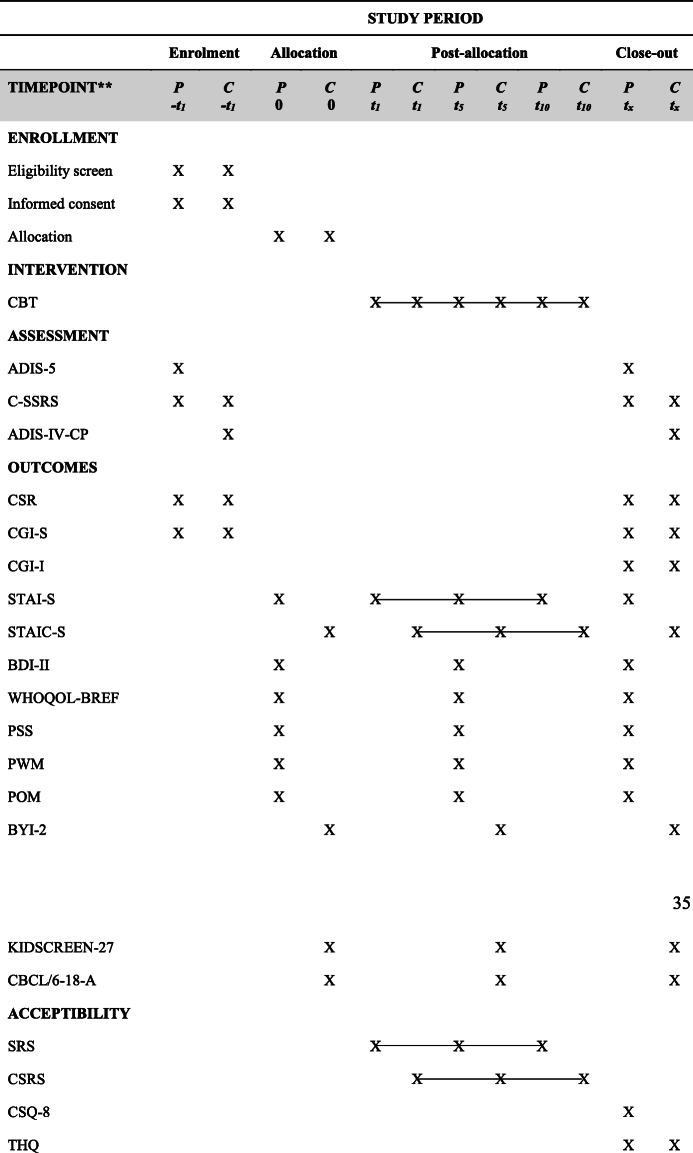

**Table 2:**
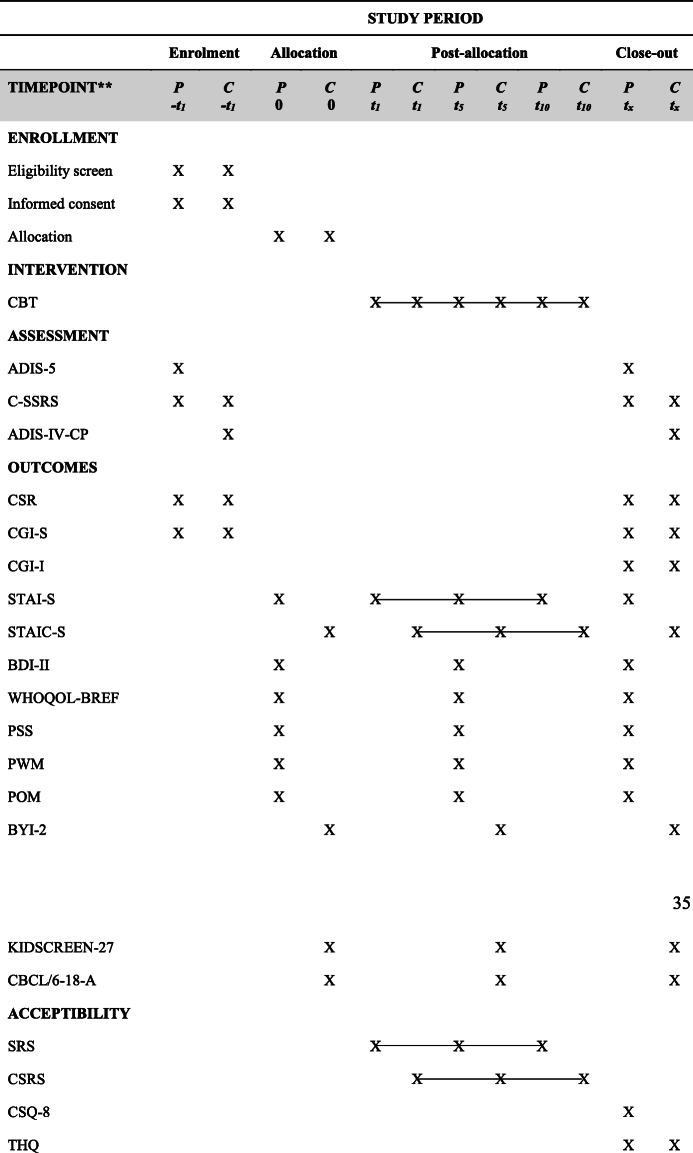
Schedule of enrolment, interventions, and assessments

Note: *P* parents, *C* child, *-t*_*1*_ pre-intervention assessment, *t*_*0*_ feedback session, *t*_*1*_ treatment session 1, *t*_*5*_ treatment session 5, *t*_*10*_ treatment session 10, *t*_*x*_ post-treatment, *X* administered at that time-point;  measured/undertaken weekly, *ADIS-5* Anxiety Disorders Interview Schedule for DSM-5, *ADIS-IV-CP* Anxiety Disorders Interview Schedule for DSM-IV Child and Parent Version, *BDI-II* Beck Depression Inventory, *BYI-2* Beck Youth Inventory, *CBCL/6-18-A* Child Behaviour Checklist Anxiety Subscale, *CBT* cognitive behavioural therapy, *CGI-I* Clinical Global Impressions Improvement, *CGI-S* Clinical Global Impressions Severity, *CSQ-8* Client Satisfaction Questionnaire, *CSR* Clinician Severity Rating, *CSRS* Child Session Rating Scale, *C-SSRS* Columbia-Suicide Severity Rating Scale, *POM* Parental Overprotection Measure, *PSS* Parental Stress Scale, *PWM* Parent Worry Measure, *SRS* Session Rating Scale, *STAIC-S* State-Trait Anxiety Inventory Child State Form, *STAI-S* State-Trait Anxiety Inventory State Form, *THQ* Therapy Helpfulness Questionnaire, *WHOQOL-BREF* World Health Organization Quality of Life Scale Abbreviated Version

#### Feasibility measures

In line with the CONSORT definition of a pilot trial [51], estimates of variability for the following outcome measures which are planned to be administered in a future RCT will be utilised in the current study. These measures will provide preliminary indications of participants’ intervention response and enable effect size calculations to inform the sample size for a future definitive RCT:

The ADIS Clinician Severity Rating (CSR) [55, 56] will be used to quantify anxiety disorder symptom severity and impairment for both parent and child participants. A CSR rating of ≥ 4 (moderate psychopathology) is considered to meet the threshold for a clinically significant diagnosis [56]. The Clinical Global Impressions (CGI) [58] will be utilised to specify parent and child participant illness severity (CGI-S) and clinical improvement or worsening (CGI-I) in comparison to baseline presentation. Participant self-report of pre- and post-treatment anxiety symptoms will be assessed using the State-Trait Anxiety Inventory child (STAIC-S) [59] and adult (STAI-S) [60] versions. Table 2 summarises all outcome measures and assessment time-points.

Fluctuations in self-reported anxiety symptom severity throughout the course of the intervention will be monitored using the STAIC-S and STAI-S. Parent ratings of child anxiety symptoms will be captured using the Child Behaviour Checklist Anxiety Subscale (CBCL/6-18-A) [61]. The Beck Depression Inventory (BDI-II) [62] and Beck Youth Inventories (BYI-2) [63] will be used to determine recent depressive symptoms in adults, and depression, anxiety, anger, disruptive behaviour, and self-concept in children. Quality of life across broad life domains will be evaluated using the World Health Organization Quality of Life Scale Abbreviated Version (WHOQOL-BREF) [64] and KIDSCREEN-27 [65]*.* Parenting-related symptoms and behaviours will be assessed with the Parental Stress Scale (PSS) [66], the Parent Worry Measure (PWM) [67], and Parental Overprotection Measure (POM) [68].

Recruitment rates will be determined by the proportion of dyads who meet the intervention eligibility criteria and consent to enter the trial. We will consider the success threshold for recruitment rates ≥ 70%. The proportion of dyads that consent and remain participants at the end of the defined study period will be used to evaluate completion rates, with ≥ 80% considered feasible. Adherence to study requirements includes dyad completion of homework tasks, as well as completion of clinician and self-report questionnaires at defined assessment time-points. For all adherence measures, the success threshold will be ≥ 80%.

#### Acceptability measures

Considered within the conceptual framework of healthcare intervention acceptability as defined by Sekhon et al. [52], participants’ acceptability of the intervention will be explored utilising the following quantitative and qualitative measures:

The Session Rating Scale child (CSRS) [69] and adult versions (SRS) [70] will be utilised to assess participants’ perspectives across key treatment dimensions. Participants’ perceptions of therapy helpfulness and credibility related to specific CBT components will be rated with the Therapy Helpfulness Feedback Questionnaire (THQ) [71]. Additionally, five open questions to elicit feedback related to the intervention experience and engaging in observational exposures are included. Child participants will complete a modified language version of the adult THQ. Parents will complete the Client Satisfaction Questionnaire (CSQ-8) [72], which will measure their satisfaction with the intervention.

Qualitative interviews will be conducted to explore participants’ prospective and retrospective acceptability. Topics of interest covered in the interview questions will be (1) perceptions of the overall enhanced intervention experience and (2) acceptability of adapted intervention components, with a focus on the novel exposures. Additionally, parents will be asked questions pertaining to (3) the bidirectional influence of anxiety between parent and child, as this relationship is considered a key conceptual underpinning of the enhanced protocol. Part 1 of the parent interview asks about their experience, while part 2 enquires about parents’ perspectives of their child’s experience. Separate versions of questions were developed contingent upon participants’ previous involvement in the intervention.

### Data collection

The diagnostic assessments, intervention, and qualitative interviews will be conducted by CS and SG, both provisional psychologists and advanced Doctor of Psychology (Clinical) post-graduate students. CS and SG have prior clinical experience in the provision of general and transdiagnostic CBT techniques. Regular oversight and supervision will be provided from two registered psychologists with extensive experience in clinical practice and research, KL and MY. In addition, an external expert in parenting and qualitative research methods will be regularly consulted throughout the iterative processes of the interview schedule development, qualitative data collection, and analysis.

Prior training will be conducted to achieve inter-rater reliability greater than 80% for ADIS diagnostic assessments. KL will randomly review 20% of diagnostic interviews and intervention videos, to provide supervision, ensure assessment and treatment fidelity, and to prevent clinician drift. To preclude the potential for clinician bias during the collection of pre- to post-intervention outcome measures, the same clinician will not deliver the intervention and conduct the diagnostic assessments for an individual client. SG will conduct the child assessments and deliver parent intervention, while CS will conduct the parent assessments and deliver child intervention.

### Data management

Participant data will be de-identified and labelled using unique alphanumeric codes. All interviews and intervention sessions will be video/audio recorded. Qualitative interviews will be transcribed verbatim using an electronic transcription programme. QSR International’s NVivo [73] will be used to store and organise qualitative study material to aid in data analysis. All study electronic data including recordings and transcriptions will be password protected and stored on a secure research drive only accessible to the study researchers. Hard-copy files will be stored in locked filing cabinets at the study site. Double data entry will be conducted for 10% of participant questionnaires to promote data quality. For the clinical trial, all raw data pertaining to parents will be stored for 7 years from the last encounter, as required by law. All raw data pertaining to child participants will be stored until the child attains the age of 25 years. For the qualitative study, all digital data collected will be permanently deleted 5 years after the last publication from this research.

### Data analysis plan

To assess feasibility related to preliminary indications of participants’ intervention response and effect size calculation, repeated-measures analyses of variance (ANOVAs) will be conducted between pre- and post-treatment for outcome measures of the CSR, CGI, STAI-S, and STAIC-S. Repeated measures ANOVAs will be utilised to analyse pre-, mid-, and post-treatment scores on outcome measures of BDI-II, WHOQOL-BREF, PSS, PWM, and POM, and the BYI-2, KIDSCREEN-27, and CBCL/6-18-A. Prior to analysis, data will be screened and relevant assumptions checked. For repeated-measures ANOVAs, partial eta-squared will be calculated as the measure of effect size. Descriptive statistics and visual inspection of scatter plots will be reported session-by-session on the STAI-S and STAIC-S. The feasibility measures of recruitment rates, completion rates, and adherence to study requirements will be determined by calculating the percentages of these measures throughout the study period.

Acceptability will be assessed utilising a mixed-methods approach. Descriptive statistics of participants’ ratings on the quantitative measures of CSQ-8, THQ, and SRS will be reported. Qualitative interviews will be analysed utilising reflexive thematic analysis [74]. Accordingly, full interview transcripts will be repeatedly reviewed for data familiarisation; initial codes will be generated, followed by collating codes into themes, refining themes, and finally defining themes and sub-themes [74]. The qualitative analysis will be undertaken utilising an iterative review process in collaboration with supervisors MY, KL, and research collaborators.

### Possible harms

Potential harms of being involved in the intervention and qualitative interviews will be explicitly outlined in the participant explanatory statements. The primary potential risk for participants is experiencing psychological distress during assessment, intervention, or interview procedures; however, this is not anticipated to exceed levels of psychological distress typically experienced in their daily lives. To minimise risk of harms, participants will be informed that they may freely withdraw their participation from any procedure at any time. Further, a range of psychological support options, including 24-h crisis support, will be discussed with participants prior to their involvement. All study clinicians and interviewers are experienced working with individuals with anxiety and other emotional problems, and in responding to distress. Adverse events to participants (e.g. significant symptom deterioration, suicidal ideation or attempt, reported or observed abuse) will be monitored routinely throughout the study. Any adverse events will be immediately reported to the principal study investigator, KL, and specific harm minimisation and prevention protocols will be enacted.

### Ethics approval and dissemination

All study procedures will be conducted in accordance with the Monash University Human Research Ethics Committee approval (project ID 9781). Study results will be disseminated through peer-reviewed scientific journals. Two publications are expected, one reporting intervention and qualitative outcomes for parents and the second reporting these outcomes for child participants.

## Discussion

This pilot study aims to investigate the feasibility and acceptability of an enhanced parent–child intervention which augments standard CBT to include treatment components targeting bidirectional maintenance factors of anxiety in parent–child dyads. In addition to evaluating the overall intervention feasibility and acceptability, this study will specifically explore participant responses to the novel intervention component of joint observational exposures. The results of this trial will inform the development and implementation of a future definitive RCT to evaluate intervention efficacy.

We anticipate that participating in the intervention will result in short-term improvements in symptomatology from pre- to post-treatment. Additionally, we expect that feasibility estimates for completion, recruitment, and adherence will be met, and results of outcome measures will enable an effect-size estimation for future RCT planning [53]. It is also anticipated that parent and child participants will prospectively and retrospectively find the overall intervention and joint observational exposures acceptable. The qualitative component provides a unique opportunity to gain a rich perspective of consumers’ experience and acceptability, to inform future planning and trialling of the enhanced intervention. The broader study implications may highlight the importance of targeting bidirectional maintenance factors in subsequent research exploring treatment of anxiety disorders in this population.

While recognising the potential implications of this research, the limitations must be acknowledged. A limitation of the proposed open-label design is that it lacks a control condition. Accordingly, we are unable to draw definitive conclusions regarding the short-term benefits of the intervention. Additionally, as a mixed-methods approach will be utilised to investigate intervention acceptability, qualitative findings cannot be generalised to a broader population. Nevertheless, despite the inherent limitations of the intended methodology, this research proposes an important initial step prior to conducting a definitive RCT [49] to determine the efficacy of the enhanced CBT intervention for anxiety in parent–child dyads.

### Trial status

This research study was prospectively registered with the Australian New Zealand Clinical Trials Registry (ANZCTR): 12619000334101. Recruitment commenced November 2019; the intervention is expected to conclude late 2020. Following completion of the pilot trial, data collection for the qualitative component will commence. Following final data collection and analysis, outcomes will be prepared for publication in 2021.

## Data Availability

The datasets generated and/or analysed during the current study will not be publicly available as consent will not be obtained from participants for this purpose.

## Abbreviations

ADIS-5: Anxiety Disorders Interview Schedule for DSM-5
ADIS-IV-CP: Anxiety Disorders Interview Schedule for DSM-IV Child and Parent Version
BDI-II: Beck Depression Inventory
BYI-2: Beck Youth Inventory
CBCL/6-18-A: Child Behaviour Checklist Anxiety Subscale
CBT: Cognitive behavioural therapy
CGI-I: Clinical Global Impressions, Improvement
CGI-S: Clinical Global Impressions, Severity
CSQ-8: Client Satisfaction Questionnaire
CSR: Clinician Severity Rating
CSRS: Child Session Rating Scale
C-SSRS: Columbia-Suicide Severity Rating Scale
DSM-5: Diagnostic and Statistical Manual, 5^th^ edition
PAM: Parental anxiety management
POM: Parental Overprotection Measure
PSS: Parental Stress Scale
PWM: Parent Worry Measure
RCT: Randomised controlled trial
SRS: Session Rating Scale
STAIC-S: State-Trait Anxiety Inventory Child State Form
STAI-S: State-Trait Anxiety Inventory State Form
THQ: Therapy Helpfulness Questionnaire
WHOQOL-BREF: World Health Organization Quality of Life Scale Abbreviated Version
